# On the Hypodermic Treatment of Uterine Pain

**Published:** 1864-10

**Authors:** J. Henry Bennet

**Affiliations:** Late Physician-Accoucheur to the Royal Free Hospital


### ON THE HYPODERMIC TREATMENT OF UTERINE PAIN.

By J. HENRY BENNET, M.D.,
Late Physician-Accoucheur to the Royal Free Hospital.

I am not aware to what extent the hypodermic injection of sedatives has been resorted to for the treatment of uterine pain, since it was first introduced to the profession, but I am desirous of giving my testimony to its extraordinary efficacy in cases presenting that symptom. I may add, that my attention was first forcibly directed to this mode of treatment by the valuable papers of Mr. Charles Hunter in *The Lancet.*

During the present winter, I have used, with prompt and marked success, the hypodermic injection in several cases of severe dysmenorrhœa, with or without hysterical complications, and several others of uterine and ovarian neuralgia, and of facial neuralgia having uterine origin. The relief has been obtained in from fifteen to thirty minutes, without being attended or followed by the headache, loss of appetite, or nausea which are so frequently theates in a result of the use of opiny other way, even by injection into the rectum. This latter mode of administering opiates has hitherto been my sheet-anchor in the treatment of uterine spasms and pain, and is certainly most efficacious; but it is not unfrequently attended by all the abovementioned drawbacks, from which the hypodermic injection appears to be singularly free. In nearly all the instances in which I have tried this mode of introducing opiates into the system, the sedative result alone has been produced; there has been no subsequent bad effect whatever.

In one case of severe uterine tormina and pain, the result of arrested menstruation from cold, I injected thirty minims of the solution of morphia. In half an hour the pains, which had been agonizing for the previous twenty-four hours, were calmed. A good night's rest followed; and the next morning the menses had resumed their course, and my patient was all but well. In another similar case, the uterine pain was accompanied by severe hysterical symptoms. The injection was followed by the same favorable result:—ease, sleep, and rapid disappearance of all morbid symtoms.

Owing to the complete control over the element of pain which the hypodermic injection of opiates appears to give, I have been able to carry on the necessary treatment, in an interesting case of uterine disease, which I should otherwise have been obliged to treat under chloroform, or at a great disadvantage. The patient, a young German lady of twenty-four, came to Mentone last autumn, by direction of her medical attendants, with the view of spending the winter in the South. She was considered to be suffering from neuralgia, facial and general, and from nervous irritability of the system in general. She had been traveling with her husband from place to place from bath to bath, in search for health, for more than two years. On being consulted, I recognized the existence of a host of uterine symptoms, and found that the neuralgic and nervous illness had manifested itself after a severe confinement, which had occurred about three years ago. The discovery of extensive inflammatory ulceration of the neck of the womb gave the key to the state of ill health. Singularly enough, none of her previous medical attendants had suspected the uterine origin of the neuralgia. Such cases are always very difficult to treat; interference with the uterine lesion all but invariably rousing the neuralgia. I have repeatedly had cases of the kind that I could only examine and treat locally, by giving chloroform to the full surgical extent on each occasion, and this I have had to do twenty or more times in the same patient.

With the patient in question the surgical treatment of the ulceration was borne tolerably well at first, but as the diseased surface became more healthy, and consequently more sensitive, endurance diminished. Every time the sore was touched, severe neuralgia followed, and the general health began to flag. In former days I should have suspended all treatment, and have sent the patient to the country for couple of months to allow the nervous system to calm down, and to let Nature do her best. In this instance such a course was not desirable, my patient being very anxious to continue the necessary treatment so as to be locally cured before we separated in the spring. I thought, therefore, of the hypodermic treatment, and tried the injection of thirty minims of the solution of morphia immediately after each uterine dressing. This course was attended with complete success; no neuralgia ensued, and I have been able to continue uninterruptedly the treatment now all but brought to a successful issue. On one occasion I omitted the precaution, and was sent for at ten o'clock at night. I found the patient a prey to a most distressing attack of facial neuralgia, which had come on an hour before. She was positively convulsed and shrieking with agony. Chlorodyne, sulphuric ether, &c., had been taken, with no relief. I injected the thirty minims of morphia solution, and in twenty minutes she was calm and free from pain. It was repeated next day, and the facial neuralgia has not returned. This lady no doubt will gradually recover her health and get rid of the neuralgia when the uterine disease is thoroughly cured.

In a case of pure neuralgia, attacking first one and then another part of the body, I have injected from twenty to thirty minims of the acetate of morphia solution, forty-two days in succession, without any unfavorable result. The neuralgia, which was very severe, was entirely subdued by it for about eighteen or twenty hours, when it re-appeared, gradually increasing in intensity until the injection again relieved it. At the end of that long period the pains gave way, the treatment having been either curative, or having allowed the neuralgic attack to wear itself out. During the entire period of treatment, the patient, a very delicate lady, slept better than usual, ate as well (her appetite being usually bad, and the digestive powers weak), and was able to take part socially in all that was going on around her. No one, indeed, was aware, except her family, that she was suffering from so painful a malady. To my surprise, I was able to suspend the morphia suddenly, without any of the distress and discomfort which is habitually observed when opiates have been long used and are abruptly abandoned.

From what I have seen of the hypodermic system, I believe that its use is capable of great extension in the treatment of pain generally. I consider that the injection of a solution of morphia after any operation would deaden pain, and produce a general calm of the system both soothing and beneficial to the patient. I think also that this result might be obtained in most cases without the usual drawbacks of opiates taken internally.

Some years ago, I recommended in this journal the injection of opium into the rectum as a means of modifying and even arresting obstinate sea-sickness. Since then various additional cases have come under my notice illustrating its efficacy. The great difficulty to all medication in sea-sickness is the fact that the stomach absorbs fluids with difficulty. By injecting subcutaneously, this difficulty is got over. Moreover, a sub-cutaneous injection would be managed easier on shipboard than the rectal injection, to which most people have a very natural antipathy.

I have used all but exclusively a solution of acetate of morphia in distilled water. Nine grains dissolved in two ounces of water gives a strength about equivalent to that of laudanum. The liquor morphiæ of the Pharmacopœia contains spirit, and and I have found that it constantly occasions small patches of painful inflammation; without the spirit, on the contrary, it appears to be quite innocuous. A moderate sized steel needle or canula I find preferable to the small gold one: The steel canula is sharper and passes easier through the skin. By pinching firmly the fold of skin that has to be pierced between the finger and thumb, its sensibility to the puncture is much diminished. It does not seem to matter much, as regards results, in which region of the body the injection takes place. I have principally chosen the præcordial region for uterine and general pain, and for local neuralgia, a spot as near the region affected as possible.

